# Mauriac Syndrome in a Child with a Positive Antinuclear Antibody Screen

**DOI:** 10.1155/2009/765318

**Published:** 2009-11-30

**Authors:** John F. Pohl

**Affiliations:** Department of Pediatric Gastroenterology, Primary Children's Hospital, University of Utah School of Medicine, Salt Lake City, UT 84113-1103, USA

## Abstract

A 17-year-old male with type 1 diabetes mellitus (T1DM) presented to clinic with elevated transaminases and a positive antinuclear antibody (ANA) screen. Due to concern for autoimmune hepatitis, a liver biopsy was performed which revealed Mauriac syndrome. This case report is the second known description of a child with Mauriac syndrome presenting with positive autoimmune markers.

## 1. Introduction

 Mauriac syndrome is associated with poor control of T1DM and presents as hepatomegaly and elevated transaminases [1]. It is typically associated with growth failure and delayed pubertal maturation, although these effects can be reversed with good glycemic control [2]. 

## 2. Case Report

 A 17-year-old male was referred to the pediatric gastroenterology clinic due to elevated transaminases noted during standard screening blood work. The patient had a history of type I diabetes mellitus (T1DM) diagnosed since 5 years of age with the patient having multiple hospital admissions for diabetic ketoacidosis secondary to noncompliance with insulin therapy. Past medical history was significant for pyloric stenosis repair at 4 weeks of age and a prior history of tonsillectomy and adenoidectomy. Family history was noncontributory. His current medication consisted of Humalog Mix 75/25 insulin (Eli Lilly and Company) with the patient receiving approximately 1.3 units of insulin per kilogram body weight. 

The patient had no history of jaundice or scleral icterus, and he denied right upper quadrant pain, pruritis, weight loss, ascites, hematemesis, or rectal bleeding. A review of his blood glucose monitoring demonstrated a range of 250–310 milligram per deciliter (mg/dL), and he had a hemoglobin A1c of 12.3% suggesting poor glycemic control. 

**Figure 1:**
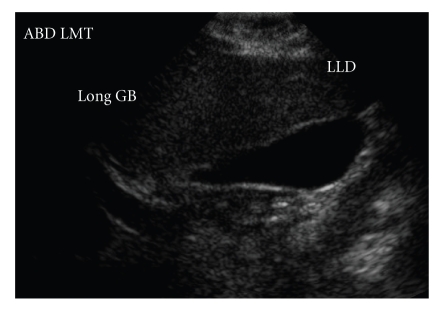


A liver ultrasound showed normal hepatic echotexture and minimal sludge in the gallbladder (Figure 1). A complete blood count, prothrombin time, activated partial thromboplastin time, lipid panel, free thyroxine level, and tissue transglutaminase serum IgA level were normal. However, transaminases were elevated with an aspartate aminotransferase (AST) and alanine aminotransferase (ALT) consisting of 63 international units per liter (IU/L) and 209 IU/L, respectively. Direct bilirubin, alkaline phosphatase, and gamma-glutamyl transpeptidase levels were normal. A viral hepatitis panel, serum ceruloplasmin, and alpha 1-antitrypsin phenotype were normal. The patient was noted to have an elevated ANA titer (1 : 160 in a homogenous pattern). Screening for smooth muscle antibody was negative, and it was decided that a percutaneous liver biopsy should be performed due to the possibility of type 1 autoimmune hepatitis. 

**Figure 2:**
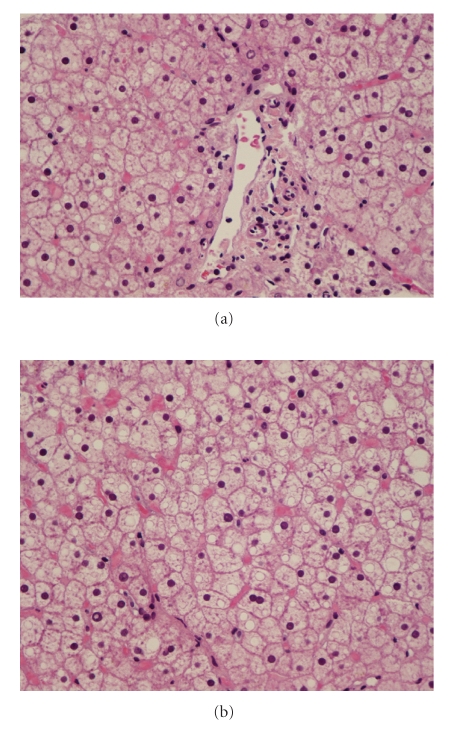


A subsequent liver biopsy demonstrated normal portal tracts (H&E 100×, Figure 2(a)); however, hepatocytes demonstrated cytoplasmic clearing secondary to increased intracellular glycogen and microvesicular fat (H&E 200×, Figure 2(b)). This biopsy was consistent with Mauriac syndrome and the importance of improved adherence to insulin therapy was expressed to the patient and his family. 

## 3. Discussion

Mauriac syndrome, first described in 1947, is a rare complication associated with T1DM and is typically associated with poor insulin compliance and glycemic control [1]. Although hepatomegaly and elevated serum transaminases are common findings in Mauriac syndrome, other described pediatric manifestations can include malnutrition, growth failure, and development of cushingoid features [1, 2]. Malnutrition associated with poor T1DM control also can lead to false elevation of the sweat chloride concentration, so such patients can present with false-positive screens for cystic fibrosis [3].

T1DM is associated with other autoimmune diseases, including celiac disease and autoimmune thyroiditis, and it is common for patients with T1DM to have elevated autoantibody titers [4]. Only one prior case report has described Mauriac syndrome in the setting of positive autoantibodies. In this case, a 16-year-old male with poorly controlled T1DM presented with elevated serum transaminases and a positive ANA of 1 : 640. A subsequent liver biopsy was consistent with Mauriac syndrome [5].

Typically, a liver biopsy in the setting of Mauriac syndrome will demonstrate steatosis as well as glycogen deposition although such findings can vary in presentation [6, 7]. Poor T1DM control leads to fatty acid transport to the liver, due to hyperglycemia and low insulin levels, which causes hepatomegaly and characteristic liver biopsy findings. These findings reverse with improved insulin control [8].

## 4. Conclusions

Mauriac syndrome is a rare complication of T1DM. Liver biopsy may be warranted in any patient with T1DM and positive autoimmune markers in order to rule out autoimmune hepatitis. Further studies are needed to help delineate patients with Mauriac syndrome from those patients with autoimmune hepatitis. 

## References

[1] Mahesh S, Karp RJ, Castells S, Quintos JB (2007). Mauriac syndrome in a 3-year-old boy. *Endocrine Practice*.

[2] Kim MS, Quintos JB (2008). Mauriac syndrome: growth failure and type 1 diabetes mellitus. *Pediatric Endocrinology Reviews*.

[3] Polack FP, Transue DJ, Belknap WM, Freij BJ, Aughton DJ (1995). Transient elevation of sweat chloride concentration in a malnourished girl with the Mauriac syndrome. *Journal of Pediatrics*.

[4] Hunger-Battefeld W, Fath K, Mandecka A (2009). Prevalence of polyglandular autoimmune syndrome in patients with diabetes mellitus type 1. *Medizinische Klinik*.

[5] Palacios CM, Vera JP, Chinchilla JF, Marco JF, Galindo MA, Ferrer LG (2004). Hypertransaminasemia in poorly-controlled type-1 diabetes mellitus. *Revista Espanola de Enfermedades Digestivas*.

[6] Dorchy H, van Vliet G, Toussaint D (1979). Mauriac syndrome: three cases with retinal angiofluorescein study. *Diabete et Metabolisme*.

[7] Lorenz G (1981). Bioptical liver changes in Mauriac syndrome. *Zentralblatt für Allgemeine Pathologie und Pathologische Anatomie*.

[8] Farrell M, Bucuvalas J, Suchy F, Sokol R, Balistreri WF (2001). Systemic disease and the liver. *Liver Disease in Children*.

